# Polyphenol-modified probiotics for the enhanced survivability, persistence and colonization of probiotic cells in the gut

**DOI:** 10.1016/j.crfs.2026.101500

**Published:** 2026-07-16

**Authors:** Rewa Rai, Marluci Palazzolli da Silva-Padilha, Wallace Yokoyama, Nitin Nitin

**Affiliations:** a Department of Food Science and Technology, University of California, Davis, CA, 95616, USA; b USDA, ARS, Albany, CA, 94710, USA; c Departamento de Engenharia de Alimentos, Universidade de São Paulo, Pirassununga, Brazil; d Department of Biological and Agricultural Engineering, University of California, Davis, CA, 95616, USA

**Keywords:** Probiotics, Polyphenols, *Lacticaseibacillus*, In-vivo persistence, Metabolic activity, Delivery

## Abstract

Probiotics and polyphenols have the potential to modulate gut health. However, gastrointestinal barriers often limit their delivery and, consequently, their biological activity. In addition, the persistence of probiotic cells in the gut, particularly in the large intestine, is crucial to their biological function. This study develops a novel combination of polyphenolic compounds and *Lacticaseibacillus* probiotic cells to enhance the delivery and persistence of probiotic cells in the gut without exogenous polymers and coatings. In this combination, the polyphenols are loaded in the probiotic cells using a negative pressure-driven infusion process. The study evaluates the enhanced survivability of polyphenol-infused probiotics during simulated *in vitro* gastrointestinal (GI) digestion, characterizes the metabolic function of the modified probiotic cells and their antagonistic activity against target pathogens, and evaluates in vivo delivery and persistence following in vivo delivery. Results showed that catechin-infused cells exhibited 10,000-fold higher viability after simulated GI digestion compared to controls and maintained their metabolic and antagonistic activities after simulated gastric treatment. Moreover, catechin-infused cells demonstrated significantly improved survivability and persistence over 48 h and enhanced colonization in the mouse gut. Overall, this study develops a novel single-cell composition, i.e, individual bacterial cells modified with the infusion of catechin, that enhances the delivery and persistence of probiotic cells in the gut.

## Highlights

• Develop single-cell composition by loading catechin into live probiotics.
• Catechin loading enhances gastrointestinal viability of probiotics by many folds.
• Catechin-loaded probiotics retain comparable metabolic and antagonistic activity.
• Catechin loading improves persistence and colonization of probiotics in mice gut.

## Graphical abstract

**Figure:**
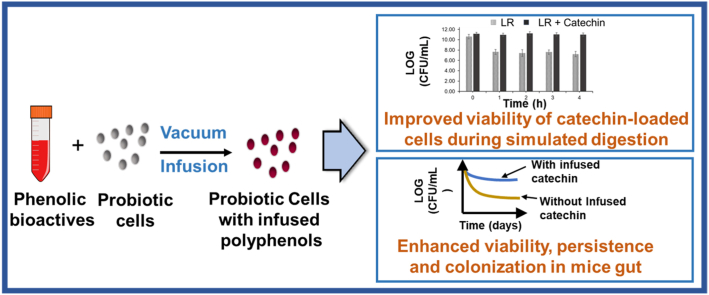

## 1 Introduction

Probiotics, polyphenols, and prebiotics have emerged as potential approaches to modulate gut health. However, the success of these approaches relies on the ability to deliver viable bacteria and adequate concentration levels of biomolecules to the gut, particularly to the large intestine, and enhance the persistence of these health-promoting bacteria in the gut (Macfarlane and Cummings, 1999; Mota de Carvalho et al., 2018; Gareau et al., 2010). The key constraint in the delivery of probiotic cells to the gut is their limited survivability in the GI tract due to the acidic pH in the gastric, bile salts, and the diversity of digestive enzymes, including proteases, lipases, and amylases in the small intestine (Macfarlane and Cummings, 1999; Gareau et al., 2010). Similar challenges of stability in the GI environment and delivery to the large intestine also exist for many polyphenolic bioactives (Sánchez-Velázquez et al., 2021; Luo et al., 2022; Rubio et al., 2014). A diversity of pharmaceutical and food-grade polymeric systems has been explored to deliver probiotics (Mota de Carvalho et al., 2018; Kim et al., 2016; Jahangirian et al., 2017). The pharmaceutical grade systems rely on conventional encapsulation of probiotics in typical non-food grade polymers such as polyvinyl alcohol, polyethylene glycol, and cellulose acetate phthalate for the gastrointestinal protection of bacteria and polyphenols (Sinha and Kumria, 2003; Charernsriwilaiwat et al., 2013; Pandian et al., 2016). However, these polymers and their combinations are not tailored to address the unique properties of vaible bacteria, including factors that may enhance the growth and persistence of these bacteria in the gut. Motivated by pharmaceutical formulations, polysaccharide-derived biopolymers such as chitosan, alginates, and functionalized starches have also been used for immobilization and delivery of probiotics (Macfarlane and Cummings, 1999; Mota de Carvalho et al., 2018; Jahangirian et al., 2017). These approaches have improved the survivability of immobilized bacteria in the GI compartments. Still, many traditional polymeric delivery systems provide narrow advantages in improving the growth and persistence of bacteria in the gut (Jahangirian et al., 2017). Furthermore, the bacteria encapsulated in a synthetic or biopolymer matrix must be adequately released to interact with the gut epithelium (Gareau et al., 2010). Similar polymeric encapsulation systems have also been developed for polyphenolic compounds (Gibis et al., 2012; Lu et al., 2025). However, there has been significantly less emphasis on modulating the delivery of these bioactives to the large intestine, and most efforts have focused on improving stability and bioaccessibility in the small intestine (Dadwal et al., 2020; Pereira Silveira et al., 2023; Vallejo-Castillo et al., 2020).

Recently, there has been an interest in developing innovative solutions using single-cell -based i.e, individual bacterial cells modified with the infusion of catechin, probiotic delivery approaches. For example, a recent study has explored using TA-nanoparticle-modified probiotics in combination with layer-by-layer biopolymer coating of single cells for enhanced IBD treatment (Zhai et al., 2024). This combination improved stability, but over 99% of the nanocoated bacteria were still inactivated after simulated gastric digestion for 2 h. Similarly, a study evaluated a nano-composite of tannic acid (TA) and casein phosphopeptide (CPP) as a probiotic coating through sequential layer technology to address the oral delivery constraints of probiotics (Qian et al., 2025). Despite the improvement with the single-cell coating approach in comparison to the non-coated cells, about 2 log CFUs of nanocoated probiotic bacteria were still inactivated after simulated gastric digestion. Inactivation of these nanocoated probiotic bacteria further increased to 3 log CFUs upon exposure to bile salts during simulated intestinal digestion. Overall, these studies highlight the potential of innovations in coating or modifying single bacterial cells to improve oral delivery. However, there is still a significant need to overcome the gastric environment and bile salt barriers. Furthermore, there is a gap in knowledge regarding the persistence of these modified bacteria for extended periods, 48 h or longer post-delivery.

Complementary to the nanocoating of single cells with polyphenol nanoparticles or polyphenolic complexes formed on the cell surface, we aim to develop a novel bio-infusion approach that combines probiotics and polyphenols to create a single-cell delivery system for enhancing the viability of delivered probiotics and their persistence in the gut (Dou et al., 2022; da Silva et al., 2023). The key advantage of this approach is the elimination of the need for exogenous biopolymers in encapsulating or coating single cells. Furthermore, this approach does not require the generation of nanoparticles and other chemical modifications of polyphenols. This novel approach using a bio-infusion of polyphenols in probiotic cells can improve the delivery and functionality of probiotics in the gut. Furthermore, this approach could potentially improve the delivery of polyphenolic compounds to the large intestine. The bio-infusion process is based on the UCD patented technology that our group has previously discovered for encapsulating lipophilic bioactives in cells based on the rapid evaporation of the carrier solvent under vacuum pressure (Nitin et al., 2020).

Thus, the central objective of this investigation was to develop and evaluate the novel bio-inspired approach of combining polyphenolic compounds with bacteria for enhancing gastrointestinal survival and delivery in vivo and to evaluate the overall persistence of this novel probiotic-polyphenolic formulation. Thus, this study evaluates the role of a model polyphenol infused in the probiotic cells on 1) the *in vitro* and in vivo survivability of the probiotic cells; 2) the metabolic activity and antagonistic activity of probiotic cells against pathogens, and 3) the *in-vivo* persistence and colonization of the probiotic cells with and without loaded polyphenols. For this, we selected catechin as the model polyphenol and *Lacticaseibacillus casei, paracasei,* and *rhamnosus* strains as the model probiotic cells. Catechin, a common flavanol in grapes and other plant-based foods, was selected for this study due to its role in modulating gut microbiota and promoting the growth of *Lacticaseibacillus* genus bacteria both in vivo and *in vitro* (Alberto et al., 2001; Gaudreau et al., 2013; Jung et al., 2019; Tzounis et al., 2011; Tabasco et al., 2011). Although not the focus of this study, prior studies have also evaluated the metabolic relationships between *Lacticaseibacillus* strains and polyphenols (Alberto et al., 2004; Wang et al., 2021). Outcomes of these studies reveal the biotransformation of polyphenolics into bioactive compounds with enhanced bioavailability and health benefits (Tabasco et al., 2011; Alberto et al., 2004; Hervert-Hernández et al., 2009; Li et al., 2013). Catechin was loaded in the probiotic cells by the negative pressure-driven loading process (Young et al., 2017). The viability of probiotic cells with and without loaded catechin was analyzed in simulated GI environments. The influence of catechin loading on metabolic and antagonistic functions of cells was assessed via resazurin-based and zone inhibition assays on agar plates, respectively. Probiotic cells with and without infused catechin were orally gavaged using a mouse model to evaluate the in vivo survivability, persistence, and colonization. Overall, this study would result in the development of novel strategies to enhance the delivery and functionality of probiotics and polyphenols in the gut.

## 2 Material and methods

### 2.1 Material

Catechin (Cayman Chemicals, Ann Arbor, USA). Guarana seed (ECRERP Cacao, Taperoá, Bahia, Brazil). Probiotic cells selected for this study include *L. paracasei* BGP-1 (Sacco, Brazil), *Lacticaseibacillus casei* (ATCC 393) and *L. rhamnosus GG* (ATCC BAA-3227)*.* Pepsin from porcine gastric mucosa (≥250 units/mg solid), calcium chloride (granular, anhydrous, ≤7.0mm, ≥93.0%), pancreatin from porcine pancreas (four × USP specifications, powder), bile salts, and monobasic potassium phosphate (≥99.0%, Sigma-Aldrich, St. Louis, MO, USA). Potasium chloride (99.7%), sodium chloride (99%), ammonium carbonate [ACS grade, ≥30.0% (NH3)], hydrochloric acid, magnesium chloride (≥99.0%), phosphate buffer saline (PBS, 10x), De Man, Rogosa and Sharpe (MRS) agar and broth, were purchased from Fisher Scientific, Pittsburgh, PA. Ultrapure water of resistivity ≥18 MΩ cm was acquired from an in-lab Milli-Q RG water ultra purification system (EMD Millipore, Billerica, MA).

### 2.2 Methods

#### 2.2.1 Vacuum infusion of catechin in probiotic cells

Probiotic cells were cultured in MRS broth (10 mL) at 37 °C for 18 h after inoculation from a frozen stock. Then, the enriched cells were further expanded in MRS broth (100 mL) to cultivate a gram of probiotic cells (∼10 log CFU) required to achieve the optimized cell-to-polyphenol concentration ratio for the infusion process. Finally, the probiotic cells were collected from the culture media by centrifugation at 5000 rpm for 5 min and washed two times with autoclaved PBS buffer.

Catechin solution was prepared by dissolving catechin in the ratio 1:5 (w/w) of probiotic cells in 25% v/v aqueous ethanol. Probiotic cells (1g) were dispersed in catechin solution by vortex mixing and placed in vacuum-sealer bags. Mixture was immediately exposed to 99% vacuum for 5 s to infuse catechin into the cells (da Silva et al., 2023). Cells without catechin served as a control. Catechin-infused and control cells were centrifuged at 5000 rpm for 5 min. Total exposure time of cells to 25% v/v aqueous ethanol during the infusion process was approximately 10 min. After centrifugation, the supernatant was discarded. Cell pellets were washed by dispersing cells in PBS buffer (1:5 w/w) using vortex mixing for 30 s. Cells were centrifuged (5000 rpm, 5min) and collected by discarding the supernatant. Cells were washed three times with PBS to remove unbound catechin. Cells (20 mg) in acidified methanol (1 mL) were mechanically disrupted using a bead beater (Bullet Blender 24, Next Advance, USA) for 30 s to aid in extracting infused catechin from cells. Cells without catechin were treated as a control. The mixture was centrifuged at 13,600 x g for 1 min. Catechin absorbance from the extracted supernatant (*λ*_max_ = 280 nm) was measured in triplicate, and concentration was quantified using the standard curve for catechin in the acidified methanol. Infusion yield (IY, mg/g of cells) and efficiency (EE, %) of catechin in cells were calculated using equations (1) and (2) respectively,

(1) $$IY\;\left(\frac{mg}{g}\right)=\frac{M_{C}}{M_{PC}}$$

and

(2) $$EE\left(\%\right)=\frac{M_{C}}{M_{T}}\times 100$$

where, *M*_*C*_ is catechin mass (mg) extracted from probiotic cells, *M*_*PC*_ is cell mass (g) used for extraction, and *M*_*T*_ is total mass of catechin (mg) used for the infusion.

#### 2.2.2 Viability of infused cells during *in vitro* digestion tests

A modified protocol suggested by Minekus et al. were used to perform *in vitro* digestion tests (Minekus et al., 2014a). Cells (1g ≈ 10 Log CFUs/mL) were dispersed in 3 mL of autoclaved water and mixed with 4 mL of simulated gastric fluid (SGF). Pepsin (2000 U/mL) was added to the digestion mixture and pH was adjusted to 3 using 1 mM of hydrochloric acid. The chemical composition of the SGF is described in Table S1. The digestion mixture was incubated in a orbital shaker (100 rpm and 37 °C) for 2 h. After the simulated gastric treatment, cells were collected from the digestion mixture by centrifugation (5000 rpm for 5 min) and transferred to 10 mL of simulated intestinal fluid (SIF, Table S1). SIF pH was adjusted to 7 using 1M sodium hydroxide. The SIF contained pancreatin (100 U/mL) and bile salts (2 mM). The SIF with the cell pellet was incubated at 37 °C and 100 rpm for 2 h for simulated intestinal digestion following the simulated gastric treatment. Aliquots (100 μL) of the digestion mixture were collected during simulated gastrointestinal digestion (t0, 1h, 2h, 3h, and 4h) to quantify the viability of cells after simulated gastrointestinal digestion. The aliquots were serially diluted, plated on MRS agar plates, and statically incubated at 37 °C for 48 h. Cells without infused catechin and cells with exogenously added catechin (without infusion) during SGF treatment were used as control samples. The simulated gastrointestinal digestion process was conducted in independent triplicate. The results of viable cell counts in log CFU/mL were reported as averages with standard deviation based on the triplicate measurements**.** Two way ANOVA followed by post-hoc Tukey HSD test was executed to evaluate significant differences among mean values of different groups. Significant differences among measurements were presented with 95% confidence interval (p < 0.05).

#### 2.2.3 Fluorescence assay for metabolic function of cells with and without loaded catechin

The resazurin reduction assay was used to assess the metabolic activity of *Lacticaseibacillus* cells with and without infused catechin and pre- and post-SGF treatment (Travnickova et al., 2019). SGF treatment was performed with and without pepsin to evaluate the pH effects on the metabolic activity of cells during SGF treatment. For this assay, cells (6 log CFU/mL) with and without catechin infusion and after 2 h treatment with SGF (pH = 3, 37 °C, 100 rpm, and with and without 3.2 mg/mL of pepsin) were suspended in 1 mL of resazurin (50 μM) added MRS broth. Cells were washed twice with 1x PBS after digestion to neutralize pH. 200 μL of each bacterial sample with added resazurin dye was added to the 96-well plate. Fluorescence intensity of resorufin (*λ*_ex_ = 530 nm and *λ*_em_ = 580 nm) generated after reduction of resazurin by the pre- and post-digested bacterial cells was measured at 37 °C after each 5 min for 24 h using a microplate reader (SPECTRAFluor Plus, Tecan SP, Inc., USA). The well plate was stirred for 10 s in a microplate reader before the acquisition of fluorescence intensity at each time point. For each sample, fluorescence intensities as a function of time were collected in triplicate to evaluate the average time for the maximum peak signal for resorufin.

#### 2.2.4 Antagonistic activity test

Antagonistic test of cells with and without loaded catechin against selected bacterial strains *Escherichia coli O157:H7* (ATCC 700728) and *Listeria innocua* (ATCC 33090) was assessed using an inhibition zone method (dos Santos et al., 2019). This test was conducted using *L. casei* as a model probiotic cell. The viable *Lacticaseibacillus* cells (10 log CFU/mL) with and without loaded catechin were spotted (4 spots each plate, 10 μL of cells per spot) on MRS agar plates. The *Lacticaseibacillus* cell solution with exogenously added catechin and catechin only was also spotted on MRS agar plates to analyze the antagonistic effect of control cells with exogenous catechin and catechin alone against target bacteria. Spotted plates were statically incubated for 24 h at 37 °C. After 24 h incubation of spot-inoculated MRS agar plates, 19 ml of BHI agar solution containing 1 mL aliquot of selected target bacterial culture (9 log CFU/mL) were poured gently over each MRS agar plates inoculated with *Lacticaseibacillus* cells. Plates were incubated for 48 h at 37 °C. The antagonistic activity was analyzed by measuring the growth inhibition zone diameter (mm) around each *Lacticaseibacillus* spot. Data were collected in triplicate for each sample and control, and measured size of growth inhibition zone were normalized dividing the diameter (mm) of growth inhibition zone by initial spot size (mm) and averaged.

#### 2.2.5 In-vivo survivability, persistence and mucoadhesion test

For in-vivo experiments using a mouse oral feeding model, *L. casei* strain was sequentially cultured in rifampicin-added MRS broth to develop a rifampicin-resistant *L. casei* strain and numbered using rifampicin-added MRS agar plates (Lee et al., 2015). Before experiment, A standard chow diet was fed to six-week-old C57BL/6J male mice (Jackson Laboratories, Sacramento, CA) for one week. For next four consecutive days, mice were fed semi-synthetic diet. Mice were food-deprived for 12 h before oral gavage. After 12 h fasting, A single dose of a rifampicin-resistant probiotic *L. casei* cells (9 log CFU) with and without loaded catechin was orally gavaged to mice. A sample size of 5 mice/group (control and test group) was chosen on the basis of a 10% body weight gain difference between mice fed test diets and mice fed the high-fat control diet from the previous studies; alpha = 0.05, beta = 0.2 and power = 0.8. The SD of C57Bl/6J mice on high-fat diets are usually around 0.7 (Seo et al., 2020). After oral gavage, mice were euthanized at 5, 24 and 48 h intervals of time to extract the contents from the small intestine, cecum, and colon. After removal of the luminal contents, Ileum, cecum, and colon tissues were also excised. All procedures were performed in accordance with institutional standard protocol approved by the IACUC of USDA-ARS-WRRC, Albany, CA. Flushed out solid contents containing cells from the small intestine were spun down by centrifugation at 5000 rpm for 5 min. In contrast, cecum and colonic contents had a high solid content and were used for analysis without centrifugation. Luminal content from each of these intestinal sections were dispersed in 5 mL of sterilized 1x PBS and each mixture was vortexed and serially diluted using 1x PBS. 100 μL of serially diluted samples and samples without any dilution were spreaded on MRS agar plates. Plates were statically incubated for 48 h at 37 °C. After 48 h of static incubation, colonies of cells on MRS agar plates were counted. Cells (CFU/mL) were calculated in each sample by multiplying the cell count on each plate by the dilution factor. The illium, cecum and colon tissue sections of mice collected after euthanization were separately soaked in 2 mL of sterilized 1x PBS and ground using a tissue grinder to remove cells from the respective tissue. The mixtures were serially diluted and instantly plated on MRS agar plates as described above. The calculated live cells (CFU/mL) in the illium, cecum and colon contents were used to evaluate the survivability and persistence and the calculated live cells in respective tissue samples were used to evaluate the colonization of cells with and without infused catechin.

#### 2.2.6 Statistical analysis

Sigma Plot v12.0 was used for the statistical analyses. Significant changes among mean values of various groups was calculated using One-way ANOVA with post-hoc Tukey HSD test. Significant changes among measurements were reported with 95% confidence interval (p < 0.05).

## 3 Results and discussion

### 3.1 Infusion of catechin into the probiotic cells

Catechin was loaded into the probiotic cells by the negative pressure-driven infusion process described in section 2.2.1. The application of negative pressure to the mixture of catechin and probiotic cells in an aqueous-ethanol solution (25% v/v) facilitated the rapid partitioning of catechin into the cellular compartments, as illustrated in our previous studies (da Silva et al., 2023; Young et al., 2017). The percentage of ethanol in the infusion mixture was chosen according to the results of probiotic cell viability after loading and the loading yield of polyphenols in probiotic cells (da Silva et al., 2023). Loading yield of catechin in the probiotic cells *L. casei*, *L. paracasei* and *L. rhamnosus* was 3.76 ± 0.16, 3.53 ± 0.18, and 3.35 ± 0.38 mg/g of the cells, respectively. The encapsulation efficiencies of catechin in these *Lacticaseibacillus* cells were ∼19%. Negative pressure-assisted infusion of plant extracts or purified polyphenols in the cells provides a higher loading yield of polyphenols in the cells and retains the viability of cells after the infusion process in comparison to the passive infusion method (da Silva et al., 2023; Young et al., 2017). The benefits of the negative pressure-drive loading process over the passive infusion are described in our previous study (da Silva et al., 2023; Young et al., 2017). The low encapsulation efficiency (∼19%) was due to the saturation capacity of the bacterial cells under experimental conditions rather than a limitation of the vacuum infusion process. The high initial catechin concentration in 25% ethanolic solutuon results in a relatively high denominator term in the encapsulation efficiency calculation, resulting in a relatively low percentage efficiency despite a high catechin loading capacity. Importantly, the vacuum infusion method achieved an encapsulation yield of 3 – 4 mg catechin per gram of bacterial cells, which is several-fold higher than catechin loading typically reported for conventional encapsulation systems (Snoussi et al., 2020). In addition, from a scalability perspective, the high encapsulation yield, rapid processing time (5 s), and elimination of additional formulation and post-processing steps required by conventional delivery systems make this approach highly favorable for large-scale applications.

Visualization of spatial distribution of infused catechin in cells using optical microscope was challenging as both the excitation and emisson wavelengths are in UV region (Du et al., 2020). However, our previous studies have demonstrated that the negative pressure-assisted infusion process enables intracellular, i.e. cytoplasmic partitioning of catechin rich guarana seed extract. In addition, some fractions of the infused polyphenols in this previous study could have been associated with cell walls. It is not possible due to limitstions of optical resolution to dileanate exclusively the fluorescence signal associated with the cytoplasmic and the cell wall fraction as fluorescence signal in confocal microscopy was observed throughout the cells, including the cytoplasm of the cells (da Silva et al., 2023). The intracellular partitioning of catechin rich guarana seed extract enhances the cell stability and influences the bio-accessibility of the loaded catechin during simulated GI digestion. The shelf-life of catechin-loaded *Lactocaseibacillus* cells and the release of infused catechin from cells during simulated GI digestion is described in our previous *in vitro* study (Dou et al., 2022; da Silva et al., 2023; Young et al., 2020; Rai et al., 2021). Results from our previous study showed that approximately 30% of infused catechin was retained within the cells after 4 h of simulated gastrointestinal digestion.

### 3.2 Cell survival under simulated gastrointestinal conditions

**Fig. 1:**
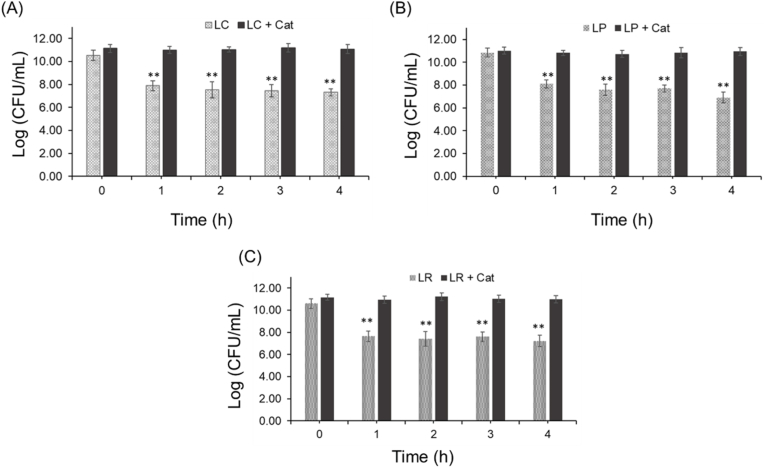
Survivability of probiotic cells (A) *L. casei* (LC), (B) *L. paracasei* (LP) and (C) *L. rhamnosius* (LR) with and without loaded catechin under simulated gastrointestinal conditions. Probiotic cells with and without loaded catechin were incubated for 2 h under simulated gastric fluid (SGF) conditions, followed by incubation under simulated intestinal fluid (SIF) for the subsequent 2 h ∗∗ indicates statistical significant difference (p < 0.01).

The substantial reduction of viable probiotic bacteria in the GI transit limits the health benefits of the orally administered probiotic cells. Survival of probiotic cell with and without loaded catechin was assessed under simulated GI conditions, as explained in section 2.2.2. Fig. 1A and B and C show the survivability (log CFU/mL) of probiotic cells *L. casei*, *L. paracasei*, and *L. rhamnosius* with and without loaded catechin during simulated digestion, respectively. Viable cell count (Log CFU/mL) of *Lacticaseibacillus* probiotic cells with infused catechin was not reduced on a log scale after 2 h treatment with the simulated gastric fluid (Fig. 1). Each selected *Lacticaseibacillus* strain, i.e., *L. casei*, *L. rhamnosius* and *L. paracasei* showed the similar trend of improved viability after simulated gastric treatment (Fig. 1A, B and C). In contrast, the viable cell count of *Lacticaseibacillus* cells without infused catechin was reduced by 2.5 log CFU/mL after treatment with the simulated gastric fluid (Fig. 1A, B and C). Cells survivability was also reduced by 2 log CFU/mL when cells were treated with simulated gastric fluid in the presence of exogeneous extracellular catechin added to the simulated gastric digestion fluid (Fig. S1). The total amount of catechin added to the simulated gastric fluid (4 mg) was similar to the total amount of catechin (∼4 mg) infused in the 1 g of cells using the vacuum infusion process. Previous studies have reported minimal impact of externally mixed polyphenols in the simulated gastric fluid for improving the viability of *Lacticaseibacillus* cells (dos Santos et al., 2019). In contrast to these results, this study illustrates a remarkable increase in the viability of cells with loaded catechin after 2h of simulated gastric treatment.

A few recent studies have investigated single-cell delivery systems using nanoscale coating or cell surface modification approaches (Zhai et al., 2024; Qian et al., 2025; Centurion et al., 2022; Fan et al., 2022). These single-cell delivery approaches are distinct from the conventional encapsulation approaches using biopolymers (Huq et al., 2013; Zhang et al., 2021; Argin et al., 2014). Some of these studies have evaluated the influence of nanocoating of specific polyphenols based on the formation of metal-polyphenol complex (Centurion et al., 2022; Fan et al., 2022; Pan et al., 2022). The results of these studies show that the nanocoating of polyphenols, such as caffeic acid and pyrocatechol, only improved the viability of probiotic cells in the simulated gastric environment by only 20% (Centurion et al., 2022) in contrast to the multi-log improvement with the loaded polyphenols in probiotic cells observed in this study. In one of these studies, it has also been shown that the coating with metal-polyphenol complexes composed of Fe (III) and different polyphenols, tannic acid, gallic acid, and epigallocatechin gallate, is pH sensitive and readily dissolves upon treatment with mild acid conditions similar to the stomach acid (da Silva et al., 2023). However, limited release of catechin (∼20% release) infused in *Lacticaseibacillus* cells was recorded after 2 h of simulated gastric digestion (da Silva et al., 2023). In addition to metal-polyphenol coatings, other encapsulation approaches, such as layer-by-layer coating of protein extract and biopolymers on probiotic cells, have also been evaluated (Sbehat et al., 2022). The results of this study indicate that layer-by-layer coating of *L. rhamnosus GG* probiotic cells with plant-extracted protein and polysaccharides, such as glucomannan, alginate, or a combination of alginate and inulin, improved the viability of cells after simulated gastric digestion by 0.5%, 5%, and 30%, respectively (Sbehat et al., 2022). Thus, illustrating a limited impact of these layer-by-layer coatings on the viability of probiotic cells under simulated gastric conditions.

In addition, this study evaluated the influence of sequential simulated intestinal digestion of the cells with and without infused catechin for 2h. The reduction in viable cell count of each selected *Lacticaseibacillus* strains, i.e., *L. casei*, *L. rhamnosius* and *L. paracasei* with infused catechin was ∼0.1 log CFU/mL after the simulated intestinal treatment following the simulated gastric treatment (Fig. 1A–C). In contrast, the viability of *L. casei*, *L. rhamnosius* and *L. paracasei* without infused catechin was decreased by 4 log CFU/mL under the simulated intestinal conditions following the simulated gastric digestion (Fig. 1A–C). This illustrates that catechin loading in cells enhanced their viability by ∼4 log CFUs during simulated intestinal digestion following gastric treatment. In comparison, probiotic cells with a nanosized surface coating composed of tannic acids and ferric ions required an additional polymeric (Eudragit L100) coating to maintain viability of probiotic cells during the simulated gastrointestinal digestion (Pan et al., 2022). In addition, few studies have co-encapsulated polyphenols and probiotic cells in biopolymer matrices for oral delivery (Shinde et al., 2014). Encapsulation of probiotic cells in biopolymer matrices enhances cell viability under simulated gastro-intestinal conditions. However, the synergistic influence of polyphenols on cell viability, achieved through co-encapsulation with probiotic cells in a biopolymer matrix, is limited (Shinde et al., 2014). These findings validate significant enhancement in the viability of *Lacticaseibacillus* cells in GI transit by loading catechin in cells.

In addition to the catechin, a plant extract isolated from guarana seeds (GSE) and reported to be a rich catechin source, was loaded in the probiotic cells by negative pressure. The method for GSE preparation and its infusion in the probiotic cells is provided in the supplementary information (SI). This extract was initially reported in our recent study on its impact on extending the shelf life of probiotics based on the infused composition. The infusion yield of GSE polyphenolics in *Lacticaseibacillus* cells was 4.5, mg GAE/g, similar to catechin's infused yield (da Silva et al., 2023). Similarly, like the infused catechin in cells, the viability of probiotic cells was not influenced by the negative pressure-driven loading of GSE in the probiotic cells (da Silva et al., 2023). In addition, cells with infused GSE also showed enhanced survivability during simulated gastrointestinal digestion compared to those without the infused extract (Fig. S2). These results further validate that the novel probiotic-polyphenolic compositions could improve the resistance of probiotics against gastrointestinal conditions, which may result in enhanced delivery of probiotics in vivo.

### 3.3 Metabolic function of catechin-loaded cells under simulated gastric conditions

**Fig. 2:**
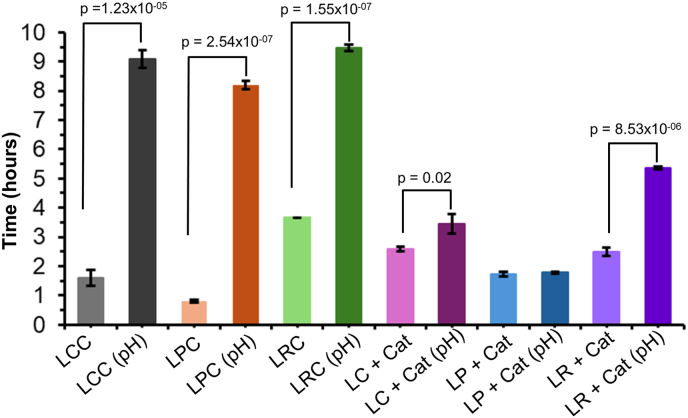
Incubation time (h) to reach maximum fluorescence intensity of resorufin (*λ*_ex_ = 530 nm/*λ*_em_ = 580 nm). Resorufin is produced by metabolizing resazurin by *L*. *casei*, *L. paracasei*, and *L. rhamnosus GG*, with and without catechin infusion and under *in vitro* gastric fluid (pH and pH + pepsin) conditions.

The metabolic function of the probiotic cells with or without loaded catechin and under the simulated gastric conditions was investigated using resazurin-based metabolic function assay. Experimental methods for the metabolic activity assays are described in section 2.2.3. The fluorescence intensity of resorufin generated by the bacterial reduction of resazurin over incubation time with the dye before and after the simulated gastric digestion is reported in Fig. S3. In this assay, higher metabolic activity and viable cell count result in a rapid reduction of resazurin and hence a rapid increase in the peak intensity, followed by a decline in the fluorescence intensity due to the generation of hydroresorufin (Labadie et al., 2021; de Oliveira et al., 2017). The incubation time period of cells with and without infusion of catechin and after simulated gastric digestion for reaching the resorufin peak fluorescence intensity is shown in Fig. 2 and Table S2. In cells infused with catechin, a shift of approximately 1 h in the time period for reaching the peak fluorescence was observed compared to the control cells. For example, the peak fluorescence of *L. casei* control cells was achieved in 1.6 ± 0.3 h, while the peak fluorescence signal for cells infused with catechin was achieved in 2.6 ± 0.1 h (Fig. 2 and Table S2). Similar trends were also observed for the *L. paracasei* and *L. rhamnosus* cells with and without loaded catechin. This suggests that the catechin-loaded cells are metabolically active, despite a small delay in achieving the peak fluorescence signal. After simulated gastric digestion, the peak fluorescence time for the control *L. casei*, *paracasei*, and rhamnosus cells was significantly delayed from 1.6 ± 0.3, 0.8 ± 0.1, and 3.7 ± 0.1 to 9.2 ± 0.2, 8.7 ± 0.1, and 8.6 ± 0.1, respectively (Fig. 2 and Table S2). This significant delay in the reduction of resazurin can be attributed to the inactivation of cells and metabolic suppression of viable cells after SGF treatment. In contrast, only a relatively smaller increase in time for the peak fluorescence signal was observed for the catechin-loaded cells after the simulated gastric digestion. The increase in time for the peak fluorescence signal for *L. casei* and *rhamnosus GG* with infused catechin was 1 and 3 h, respectively, after simulated gastric conditions treatment. The time for peak fluorescence signals of *L. paracasei* with infused catechin was not significantly (p = 0.52) changed before and after the SGF treatment (Fig. 2 and Table S2). This overall trend demonstrates that *Lacticaseibacillus* cells maintain their metabolic activity after the infusion of catechin*.* There was only a limited reduction in the metabolic function of probiotic cells infused with catechin during the simulated gastric digestion compared to those without infused catechin. However, the reduction in the metabolic function of cells under simulated gastric fluid condition in the presence of exogenous extracellular catechin added to the gastric fluid was similar to that of the cells without loaded catechin after simulated gastric treatment (Fig. S4). In a previous study, the bacterial cells coated with metal-polyphenol complexes composed of Fe(III) and tannic acid have been reported to exhibit delayed growth both after coating and following exposure to the simulated gastric fluid, indicating a change in metabolic activity (Fan et al., 2022). These evidences further support the results that the intracellular infusion of catechin has a significant role in enhancing the gastrointestinal viability of the probiotic cells while maintaining the metabolic activity of the cells. Among the SGF components, the acidic pH had a dominant role in influencing the peak fluorescence time compared to pepsin (Table S2). This verifies that the metabolic activity of cells were mainly influenced by the acidic pH of SGF.

### 3.4 Antagonistic activity of catechin-infused cells against pathogens

**Fig. 3:**
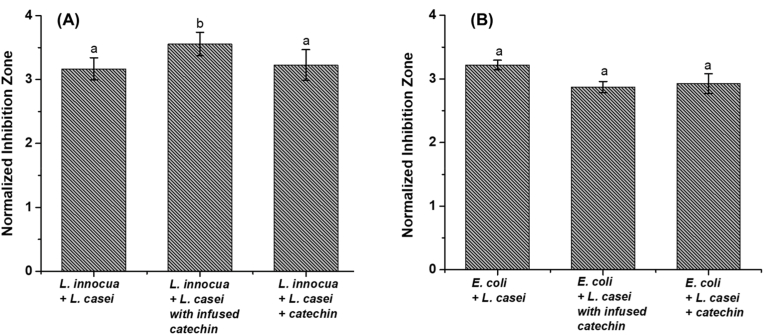
Inhibition zone assay to assess inhibitory properties of *L. casei* with and without infused catechin against *L. innocua* (A) and *E. coli* (B). The controls also include *L. casei + exogenously* added catechin.

Probiotics, especially *Lacticaseibacillus spp.* have the potential to inhibit the growth of bacterial pathogens (Karska-Wysocki et al., 2010; Chen et al., 2019; Soltani et al., 2022). Antagonistic activity of *L. casei* cells with and without loaded catechin against the gram-positive and gram-negative strains of *Listeria innocua* and *E. coli 0157:H7* strain was evaluated using the inhibition zone method described in Section 2.2.4. Antagonistic effect of *L. casei* with externally added catechin and catechin without cells were also evaluated as controls. Normalized inhibition zone and images of agar plates are presented in Fig. 3 and Fig. S5, respectively. Infusion of catechin in *L. casei* results in a slight but statistically significant increase in inhibition of *Listeria innocua* cells (Fig. 3). The inhibition zone for *E. coli* cells was not influenced by the infusion of catechin in cells. Furthermore, supplementation by physically mixing catechin with *L. casei* resulted in no significant effect on the inhibition zone (Fig. S5). These results demonstrate that *Lacticaseibacillus* cells with infused catechin show similar or improved antagonistic properties in comparison to the control cells. The improved anatagonistic properties of cells with infused catechin may be attributed to 1) although catechin alone did not exhibit significant antimicrobial activity at the tested concentration, its incorporation into the bacterial cells may have resulted in a localized and sustained release of catechin at the probiotic cell-pathogen interface, and 2) the combined presence of probiotic cells and infused catechin may have produced additive or synergistic effects through multiple mechanisms, including organic acid production, competitive interactions, and increased membrane permeability induced by catechin (López de Felipe and Laranjo, 2024; López de Felipe et al., 2022). Therefore, the enhanced inhibition is likely attributable to the combined action of the probiotic carrier and infused catechin rather than the antimicrobial activity of free catechin alone. Further studies are currently in progress to elucidate the molecular and cellular mechanisms underlying this enhanced antimicrobial activity.

### 3.5 Survivability, persistence, and colonization of probiotic cells in a mouse model

**Fig. 4:**
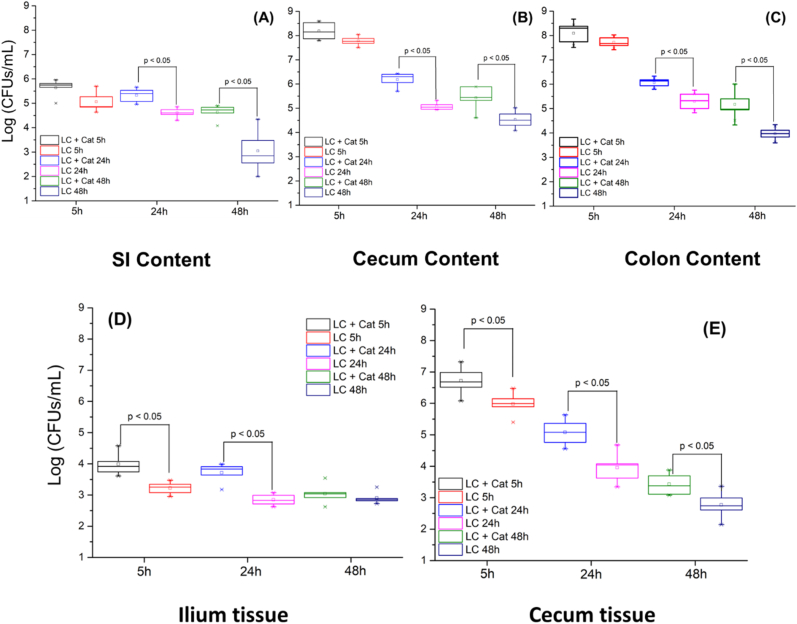
Survivability and persistence (up to 48 h) of model probiotic cell *l. casei* with and without loaded catechin in (A) SI contents, (B) cecum contents and (C) colon contents collected from the mouse GI tract. Colonization of cells with and without infused catechin in (D) ilium tissue and (E) cecum tissue. For colonization, cells attached to each tissue were extracted by grinding tissue using a tissue grinder and were enumerated after spreading serially diluted solutions on MRS agar plates.

The *in-vivo* survivability, persistence, and colonization of cells with and without loaded catechin were evaluated using a mouse oral feeding study as described in the method section 2.2.5. Fig. 4(A–C) presents that in the lumen of the small intestine, the viable probiotic cell count was 6 log CFU/mL after 5 h and in the cecum and colon, the viable probiotic cell counts were more than 8.5 log CFU/mL. The higher population of the delivered probiotics in the cecum and colon compared to the small intestine after feeding probiotic cells to the mice agrees with previous studies, including Taverniti et al. (Taverniti et al., 2021; Aktas et al., 2015). The lower population of viable probiotic cells in the small intestine is primarily attributed to their exposure to the harsh biochemical environment of the intestine, such as bile salts and digestive enzymes (Jensen et al., 2023). High log CFU/mL in the cecum and colon section contents after 5 h shows that 5 h was an adequate time for probiotic cells to reach the large intestine in a mouse model. After 5 h of orally gavaged cells (9 log CFUs/mouse), log CFU/mL of the surviving cells with and without loaded catechin were not significantly different in the small intestine, cecum, and colon, respectively. This finding was different from the *in-vitro* survivability results of probiotic cells with and without infused catechin obtained after 5 h of simulated gastrointestinal digestion.

Simulated gastrointestinal digestion for 5 h showed a significant difference between the survivability of cells with and without infused catechin. The difference could result from variations in the composition of gastric and intestinal fluids between *in vitro* and in vivo experiments. In the *in vitro* experiments, the biochemical components and their concentration levels were selected to simulate the human digestive environment (Minekus et al., 2014b). These simulated conditions can differ from the mouse model's gastric and intestinal conditions (Wos-Oxley et al., 2012). Second, during the simulated gastrointestinal digestion, the bacteria were uniformly exposed to the same gastrointestinal conditions of pH, bile salts, and enzymes for the entire duration of the simulated digestion experiment. However, during the *in-vivo* process, a more dynamic environment exists in which cells may experience a gradient of pH conditions in the gastric environment or spatial and temporal variation in bile salt levels during their gastrointestinal transit (Bohn et al., 2018). Furthermore, probiotics were administered to mice by oral gavage as a liquid formulation, with cells dispersed in sterile 1× PBS. Consequently, the formulation is expected to transit the gastric environment in overnight fasted mice more rapidly with a half life of a few minutes than a few hours of residence time for solid or semi-solid food matrices. Because liquid formulations are expected to pass through the empty stomach more rapidly, the in vivo exposure to gastric conditions is likely shorter than that simulated *in vitro*. For example, in the fasted human stomach, a water dose of approximately 240–800 mL empties rapidly, with a gastric half-emptying time ranging from about 8 to 18 min (Mudie et al., 2014). In fasted mice, the gastric half-emptying time for water is approximately 3 to 15 min (Miller et al., 2018). This in vivo condition differs from the 2 h static simulated gastric digestion used in our *in vitro* experiments, which was designed to mimic the gastric residence time of solid or semi-solid food contents rather than liquid formulations (Bennink et al., 2003; Cappello et al., 2000). Therefore, the lack of a significant difference observed after 5 h in mice may be attributed, at least in part, to the faster gastric transit of the liquid formulation delivered using an oral gavage.

However, the *in-vivo* survivability and persistence of cells with and without infused catechin in the mouse model were significantly different after 24 and 48 h. In the gastrointestinal contents isolated from the small intestine, cecum, and colon (Fig. 4A–C), the viable count of *L. casei* cells with infused catechin was at least 1 log CFU/mL higher than the viable count of cells without catechin after 48 h. This level of increased persistence of cells in the GI contents with catechin-infused probiotic cells is similar to the level of increase observed for the probiotic cells nanocoated with tannic acids using Fe(III) ion in the GI content collected after 2 days of in vivo delivery (Centurion et al., 2022) However, in this previous study with coated probiotics, the mice were orally gavaged a daily dosage of probiotic cells for a period of 6 days, and then a significant difference was observed after 48 h of the last feed. In contrast, only a single dose of probiotic cells was given in this study to attain a comparable level of improved persistence after 48 h of the first feed.

Furthermore, this study also evaluated the association of the delivered cells with the ilium and cecum tissue. For this measurement, the tissue sections were isolated and homogenized before enumerating the viable *L. casei* cells using the agar plate assay described in the methods section 2.2.5. The results show enhanced attachment of the catechin-infused *L. casei* cells compared to the control cells after 24 h of initial feeding. This difference in the viable count of cells attached to the tissue diminished over the 48 h period (Fig. 4D). In the cecum tissue, the attachment of the bacterial cells with infused catechin was 1 log CFU/mL higher than the control cells without catechin over 48 h period (Fig. 4E). This result suggests that the cell with infused catechin may have enhanced mucoadhesion properties and/or improved association with the cells of the gut tissue.

Overall, the *in-vitro* and *in-vivo* results of this study establishes that the negative vacuum-driven loading of catechin in *Lacticaseibacillus* cells has significant potential to enhance cell survival during gastrointestinal digestion and to improve persistence and colonization of cells in the gut of a mouse model. The potential mechanisms for the enhanced survivability of cells following the infusion of polyphenolic compounds are not yet fully understood, and ongoing studies are being conducted to investigate this further. However, during gastric treatment, acidic flux is attributed as one of the major causes for the reduction in the survivability of probiotic cells, in addition to the ionic strength and pepsin influence on cellular composition and structure (Yao et al., 2020; Han et al., 2021; Siegumfeldt et al., 2000). Based on the studies that have explored the mechanism of acid tolerance of probiotics, it is attributed that the availability of various hydrogen bonding groups in intracellularly distributed phenolic bioactives may influence the pH regulatory pathways or buffering capacity of cells (Corcoran et al., 2005).

In addition, *Lactobacilli* are known to tolerate relatively high concentrations of polyphenols through unique cell-surface structures, including exopolysaccharides (EPS), teichoic acids, and other cell wall-associated components that can interact with phenolic compounds (Nakayama et al., 2015). Exposure to polyphenols has been reported to induce molecular chaperones (DnaK, DnaJ, GrpE, GroEL, and GroES), ATP-dependent proteases (ClpP, ClpC, and ClpE), and heat- and alkaline-shock proteins that repair damaged proteins and maintain protein folding under stress conditions. Because acid stress can cause protein denaturation and misfolding, activation of these protective systems by catechin may enhance probiotic survival through cross-protection mechanisms (Celebioglu et al., 2018; Koponen et al., 2012).

Phenolic compounds have also been shown to influence membrane and cell-wall architecture by altering fatty acid composition, phospholipid biosynthesis, peptidoglycan synthesis, and wall teichoic acid composition (López de Felipe et al., 2022; Broadbent et al., 2010; Jordan et al., 2008). Such structural adaptations have previously been associated with improved acid tolerance in *Lactobacilli* and may contribute to the enhanced resistance of catechin-treated cells to simulated gastrointestinal conditions. Further, polyphenols can activate oxidative-stress defense pathways, which may indirectly improve tolerance to acid stress because acidic environments often promote reactive oxygen species formation and oxidative damage (López de Felipe et al., 2022). Together, these mechanisms may complement intracellular pH regulation pathways and contribute to the improved viability and functionality of catechin-treated probiotics.

Similarly, during intestinal digestion, the enhanced bile tolerance of *Lacticaseibacillus* cells with infused phenolic bioactives may be because of changes in the chemical composition and the surface properties of the cell-wall and membranes. These changes may be physical changes like surface hydrophobicity, physicochemical changes like acid and bile interaction sites at the molecular level or biochemical stress response, such as production of surface proteins or bile salt hydrolases (López de Felipe et al., 2022; Lee et al., 2008; Koskenniemi et al., 2011; Ruiz et al., 2007; Papadimitriou et al., 2016). For example, catechin infused in cell membrane can associate with phospholipid bilayers and membrane proteins through non-covalent interactions, reinforcing membrane integrity and stabilizing transmembrane protein complexes (López de Felipe et al., 2022; Nakayama et al., 2015; Sirk et al., 2008; Kumazawa et al., 2004). This protective effect may reduce bile-induced membrane damage and permeability. Similarly, these modified compositions can stabilize key stress-response proteins and can reduce oxidative modifications of stress-response enzymes by scavenging reactive oxygen species (Lee et al., 2008; Koskenniemi et al., 2011). By preserving the functionality of proteins involved in detoxification, repair, and energy metabolism, infused catechin may protect cells from bile and oxidative stresses (Ruiz et al., 2013). These changes may also influence the physicochemical interactions between cells with infused catechin and the mucosal environment (Park and Robinson, 1987; Gómez-Guillén and Montero, 2021; He et al.). Although direct molecular evidence for the stabilization of specific membrane and stress-response proteins by polyphenols remains limited, these interactions provide a plausible mechanistic explanation for the improved probiotic viability observed in catechin infused cells and future investigations based on biochemical assays, metabolomics, metagenomics, gene expressions, and transcriptomics will be conducted using different strains of probiotic cells and polyphenols.

## 4 Conclusion

This study developed a novel approach to successfully deliver the single-cell composition of probiotic cells across GI barriers and improve the persistence of these bacteria in vivo. These novel single-cell compositions were developed by infusing polyphenolic compounds, such as catechin, into probiotic cells using a negative-pressure-assisted infusion process. These novel compositions differ from conventional encapsulation approaches using polymers to encapsulate probiotic cells and some recent single-cell coating studies. The results of this study demonstrate that this novel composition improved the gastric pH and bile salt tolerance of the probiotic cells during simulated *in vitro* digestion, resulting in a several-fold increase in cell viability after simulated digestion. The results also demonstrate that polyphenol-infused probiotic cells maintained their metabolic activity and antagonistic properties against pathogens. The results of in vivo delivery demonstrate a significant enhancement in the persistence of probiotic cells in the lumen of the cecum and colon, as well as improved binding of the modified probiotic cells to the ileum and cecum tissue. Overall, the study has developed a novel approach for the improved delivery and persistence of probiotics using polyphenol-infused probiotics. Future investigations are needed to evaluate the biological activity of these probiotics in vivo and the biological factors for enhanced persistence of probiotics in the gut.

## Author contributions

Conceptualization, R.R., M.P.S. and N.N.; Methodology, R.R., M.P.S., W.Y. and N.N.; Software, R.R. and M.P.S.; Formal Analysis, R.R. and M.P.S.; Investigation, R.R. and M.P.S.; Resources, N.N. and W.Y.; Data Curation, R.R. and M.P.S.; Writing – Original Draft Preparation, R.R.; Writing – Review & Editing, R.R., M.P.S. and N.N.; Visualization, R.R., M.P.S. and N.N.; Supervision, N.N.; Project Administration, N.N.; Funding Acquisition, R.R., W.Y. and N.N.

## Declaration of competing interest

The authors declare the following financial interests/personal relationships which may be considered as potential competing interests: Nitin Nitin, Rewa Rai and Marluci Palazzolli have a patent application titled Polyphenol infused probiotics and methods for improved gut survivability, persistence and colonization pending to THE REGENTS OF THE UNIVERSITY OF CALIFORNIA, Assignors: RAI, Rewa, NITIN, NITIN, PALAZZOLLI DA SILVA PADILHA, MARLUCI. If there are other authors, they declare that they have no known competing financial interests or personal relationships that could have appeared to influence the work reported in this paper.
