# Supplementary material for: Polyphenol-modified probiotics for the enhanced survivability, persistence and colonization of probiotic cells in the gut
Source: Curr Res Food Sci. 2026 Jul 16;13:101500. doi: 10.1016/j.crfs.2026.101500 (PMC13396922; doi:10.1016/j.crfs.2026.101500)
Supplement: Multimedia component 1 [file mmc1.docx]

**Supplementary Information**

***Recovery of phenolic compounds from guarana seed and infusion in probiotic cells.*** Guarana seed extract (GSE) was produced as suggested by Silva et al. (2019) with some modifications. Dried guarana seed was added in 25% ethanol solution in the proportion 1:20 (w:w) and mixed using a mechanical stirring at 60 ºC and 200 rpm for 30 min. Mixtures were centrifuged at 9000 rpm for 5 min. Supernatants containing guarana seed extract (GSE) were collected and added to 1g pellet of each probiotic cells. Cells were dispersed in aqueous ethanol mixture of GSE by vortex mixing and exposed to vacuum for negative pressure-assisted infusion of GSE into the cells as described in our previous report by da Silva et al., 2023.

**Table S1.** Composition of stock solutions and simulated gastrointestinal fluids in 1L of double distilled water.

| Constituent | Stock | SGF | SIF |
| --- | --- | --- | --- |
| KCl | 0.5 M | 6.9 mM | 6.8 mM |
| KH_2_PO_4_ | 0.5 M | 0.9 mM | 0.8 mM |
| NaHCO_3_ | 1 M | 25 mM | 85 mM |
| NaCl | 2 M | 47.2 mM | 38.4 Mm |
| MgCl_2_(H_2_O)_6_ | 0.15 M | 0.1 Mm | 0.33 mM |
| (NH_4_)_2_CO_3_ | 0.5 M | 0.5 mM | - |
| CaCl_2_(H_2_O)_2_ | 0.3 M | 1.5 mM | 0.6 mM |

**Table S2.** Time (h) to reach maximum fluorescence intensity of resorufin, indicating metabolic activity of *L. casei*, *L. paracasei*, and *L. rhamnosus GG*, with and without catechin infusion, before and after simulated gastric treatment with or without pepsin. (*) represents the statistically significant difference between cells (with or without infused catechin) before and after simulated gastric fluid treatments. Letter ^a^ represents statistically significant difference between cells treated with SGF with and without pepsin.

| **Cells** | **Treatment Conditions** | **Time at Max Resorufin Fluorescence Intensity (hours)** |
| --- | --- | --- |
| ***L. Casei* Control (LCC)** | No treatment | 1.6 ± 0.3 |
|  | Gastric Fluid (pH 3) | 9.1 ± 0.3* |
|  | Gastric Fluid (pH 3 + pepsin) | 9.2 ± 0.2* |
| ***LC* + Catechin** | No treatment | 2.6 ± 0.1 |
|  | Gastric Fluid (pH 3) | 3.4 ± 0.3* |
|  | Gastric Fluid (pH 3 + pepsin) | 3.5 ± 0.8 |
| ***L. Paracasei* Control (LPC)** | No treatment | 0.8 ± 0.1 |
|  | Gastric Fluid (pH 3) | 8.2 ± 0.2* |
|  | Gastric Fluid (pH 3 + pepsin) | 8.5 ± 0.3* |
| ***LP* + Catechin** | No treatment | 1.7 ± 0.1 |
|  | Gastric Fluid (pH 3) | 3.4 ± 0.3 |
|  | Gastric Fluid (pH 3 + pepsin) | 3.5 ± 0.8 |
| ***L. Rhamnosus GG* Control (LRC)** | No treatment | 3.7 ± 0.1 |
|  | Gastric Fluid (pH 3) | 9.5 ± 0.1* |
|  | Gastric Fluid (pH 3 + pepsin) | 8.6 ± 0.1*^,a^ |
| ***LR* + Catechin** | No treatment | 2.5 ± 0.1 |
|  | Gastric Fluid (pH 3) | 5.4 ± 0.1* |
|  | Gastric Fluid (pH 3 + pepsin) | 6.0 ± 0.1*^,a^ |


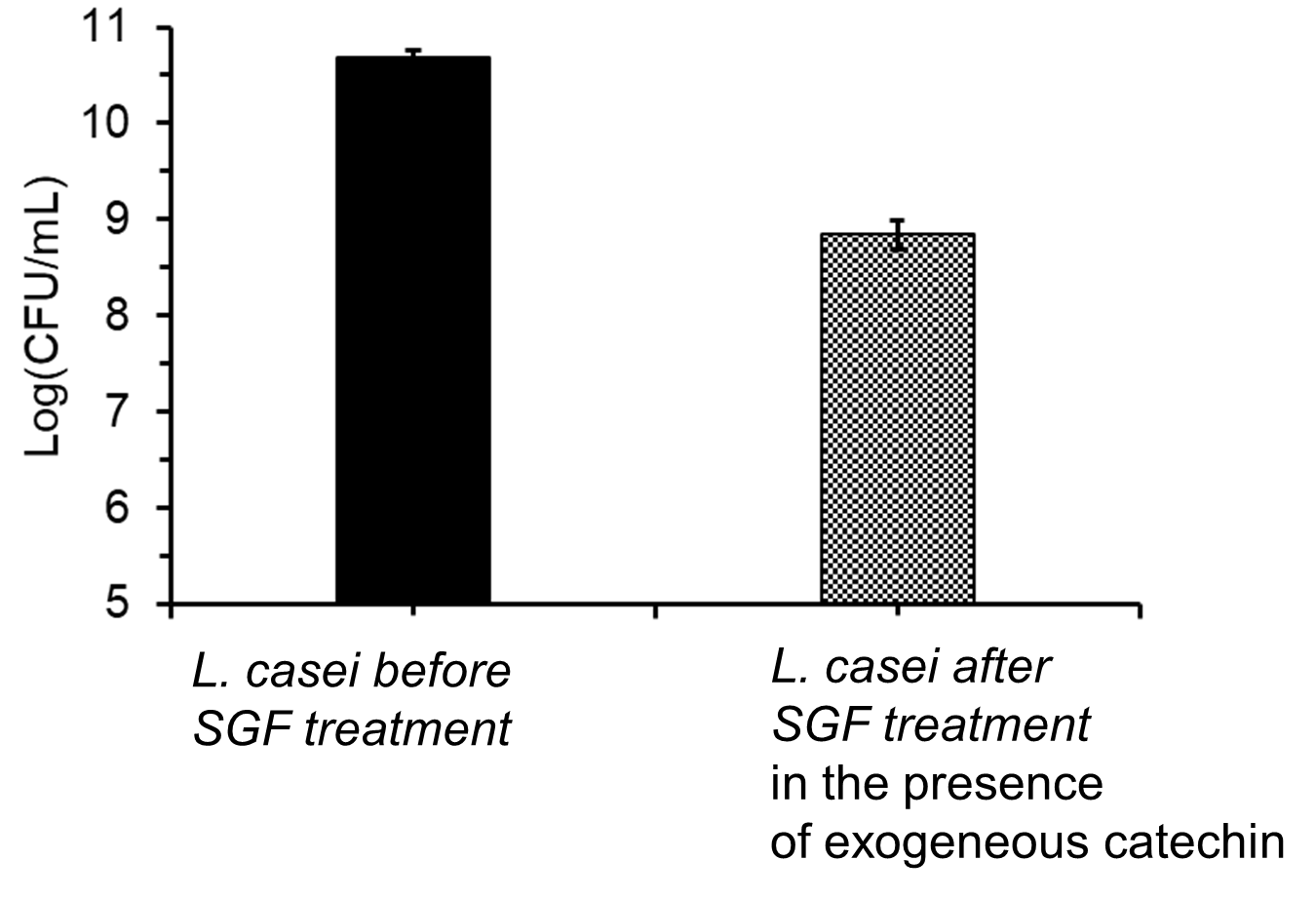


**Fig. S1**. Survivability of *L. casei* cells before and after simulated gastrointestinal digestion in the presence of exogeneous catechin.

**
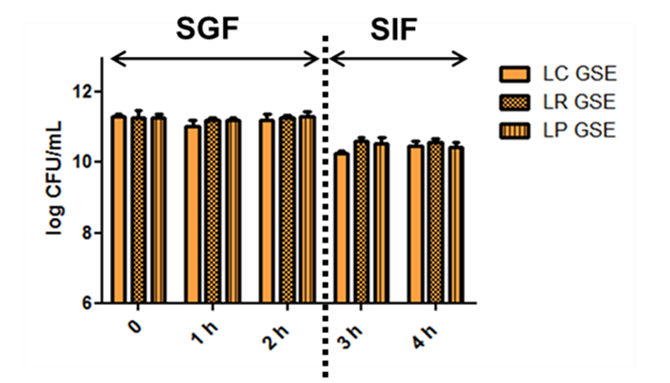
**

**Fig. S2.** Survivability of probiotic cells with infused Guarana seed extract (GSE) during simulated gastrointestinal treatments. Probiotic cells with infused GSE were treated with simulated gastric fluid (SGF) for 2h, followed by the treatment with simulated intestinal fluid (SIF) for the next 2h.


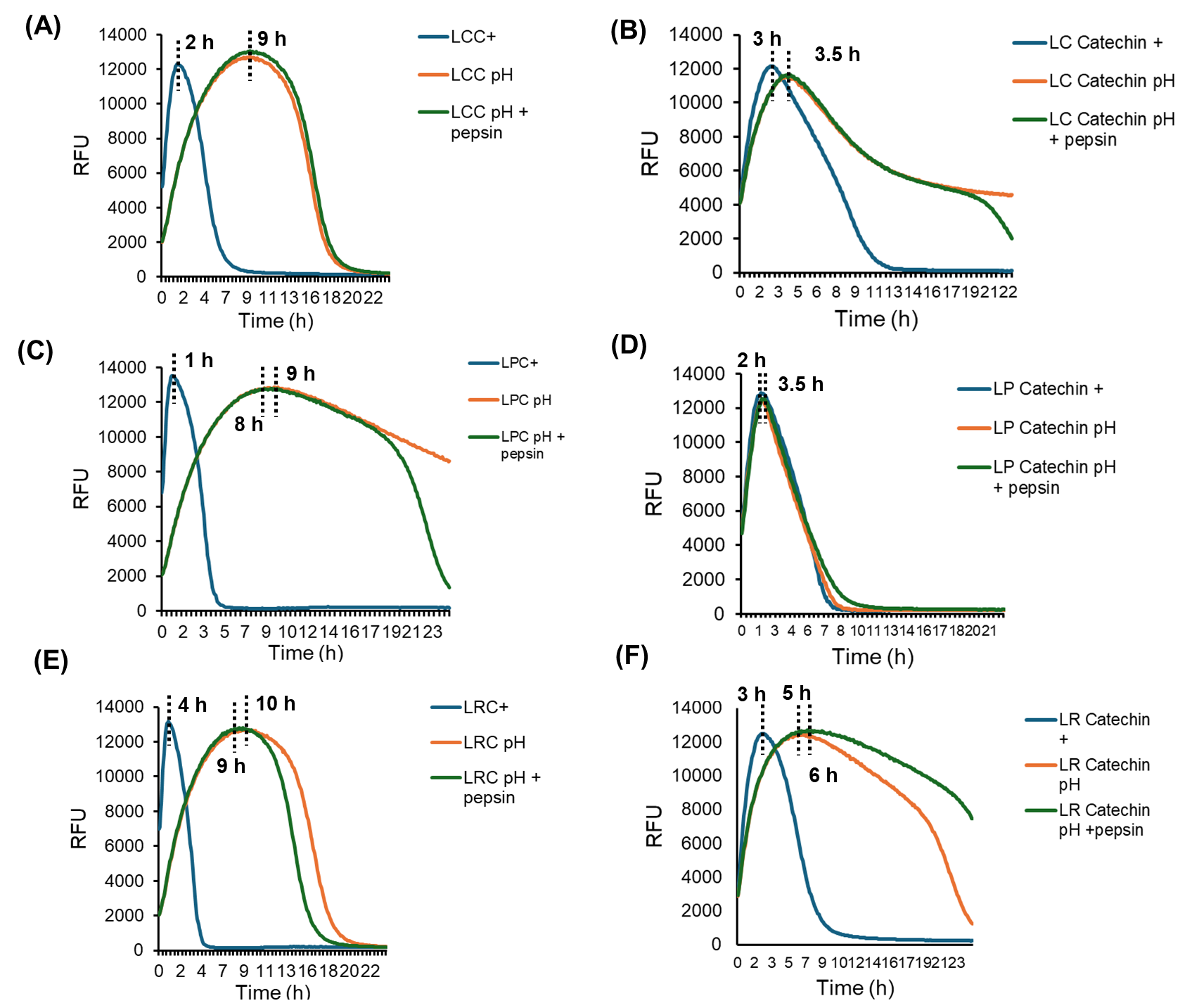


**Fig. S3.** Changes in metabolic activity of bacteria with and without catechin infusion and the influence of the simulated gastric digestion. Changes in fluorescence intensity of resorufin (*λ*_ex_ = 530 nm/*λ*_em_ = 580 nm) as a function of incubation time (h). Resorufin is produced by metabolizing resazurin by *L. casei*, *L. paracasei* and *L. rhamnosus* with (B, D and F) and without infused catechin (A, B and C) after *in vitro* treatment with gastric fluid (pH and pH + pepsin).


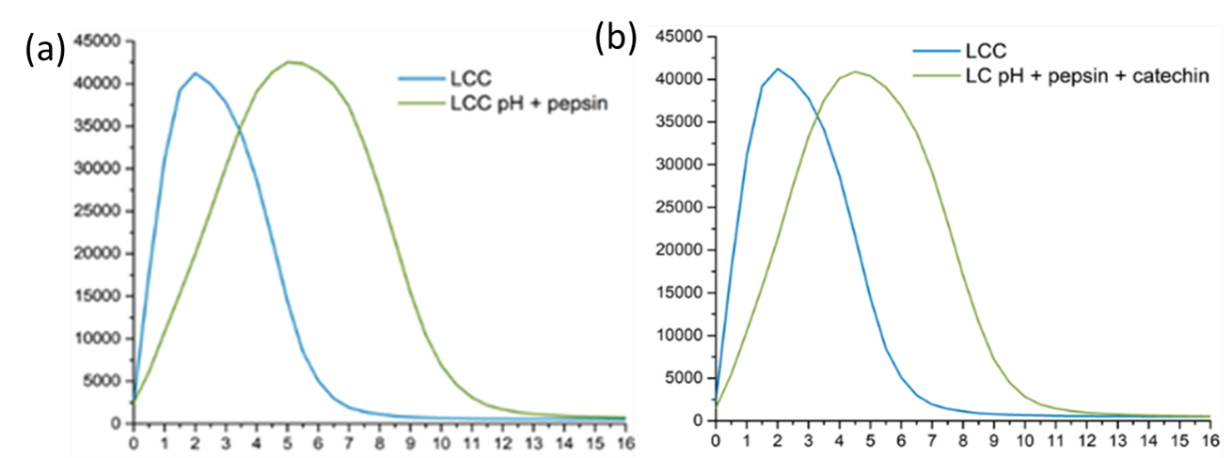


**Fig. S4.** Fluorescence intensity of resorufin (*λ*_ex_ = 530 nm/*λ*_em_ = 580 nm) against time during resazurin metabolism assay. Resorufin is produced by metabolizing resazurin by *L. casei* before and after *in vitro* gastric fluid in presence of exogeneous catechin.


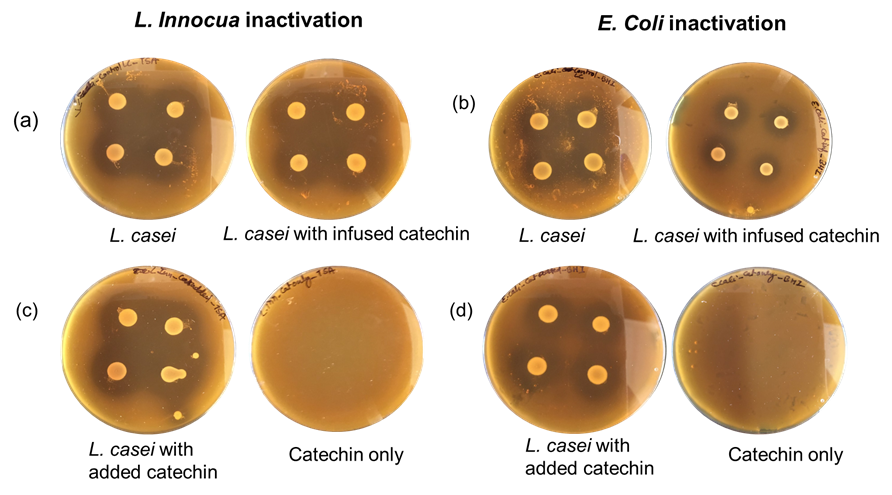


**Fig. S5.** Images of agar plates showing antagonistic activity of model probiotic cells *L.casei* with and without infused catechin (a and b) and *L.casei* in presence of exogeneous catechin and catechin without cells (c and d) against pathogens *L. innocua* and *E. coli* cells, respectively.
